# Proteomics in the Study of Bacterial Keratitis

**DOI:** 10.3390/proteomes3040496

**Published:** 2015-12-14

**Authors:** Rachida Bouhenni, Jeffrey Dunmire, Theresa Rowe, James Bates

**Affiliations:** Ophthalmology Department, Summa Health System, 525 East Market St, 274 Old Medical Building, Akron, OH 44304, USA; E-Mails: dunmirej@summahealth.org (J.D.); Theresa Rowe and (T.R.); batesj@summahealth.org (J.B.)

**Keywords:** keratitis, proteomics, bacteria, ELISA, western blotting, antibody arrays

## Abstract

Bacterial keratitis is a serious ocular infection that can cause severe visual loss if treatment is not initiated at an early stage. It is most commonly caused by *Staphylococcus aureus, Pseudomonas aeruginosa, Streptococcus pneumoniae,* or *Serratia* species. Depending on the invading organism, bacterial keratitis can progress rapidly, leading to corneal destruction and potential blindness. Common risk factors for bacterial keratitis include contact lens wear, ocular trauma, ocular surface disease, ocular surgery, lid deformity, chronic use of topical steroids, contaminated ocular medications or solutions, and systemic immunosuppression. The pathogenesis of bacterial keratitis, which depends on the bacterium-host interaction and the virulence of the invading bacterium, is complicated and not completely understood. This review highlights some of the proteomic technologies that have been used to identify virulence factors and the host response to infections of bacterial keratitis in order to understand the disease process and develop improved methods of diagnosis and treatment. Although work in this field is not abundant, proteomic technologies have provided valuable information toward our current knowledge of bacterial keratitis. More studies using global proteomic approaches are warranted because it is an important tool to identify novel targets for intervention and prevention of corneal damage caused by these virulent microorganisms.

## 1. Introduction

Infectious keratitis is a serious, sight-threatening ocular condition. Early diagnosis, identification of the etiologic organism, and prompt antimicrobial therapy are required for successful treatment. Infectious keratitis is characterized by corneal inflammation and can be caused by bacteria, fungi, viruses, or parasites [1] with bacteria causing the most threatening condition [2]. The incidence of corneal infections continues to rise proportionately with the increased use of contact lenses across the globe. Despite advances in clinical diagnosis, laboratory investigations, and the availability of potent antibiotics, visual morbidity remains high in underdeveloped countries. The prevalence of infectious keratitis ranges in different regions of the world from 6.3 to 710 cases per 100,000 individuals per year, with increased incidence among contact lens wearers [3].

Bacterial keratitis is caused by a variety of species including *Staphylococcus aureus* (*S. aureus*), *Pseudomonas aeruginosa* (*P. aeruginosa*), *Streptococcus pneumoniae* (*S. pneumoniae*) and *Serratia* species with *P. aeruginosa* being the most commonly isolated Gram-negative organism (40%–70%) [4], followed by *Serratia marcescens* (*S. marcescens*) among contact lens wearers [5]. Possible sources of these bacteria could be environmental, the patient’s normal flora, ocular devices, contact lens care solutions/cases, or topical drug/ irrigation solutions. Typical findings associated with bacterial keratitis include pain, presence of anterior chamber reaction or hypopyon, poor vision, and corneal ulcer [6]. Bacterial keratitis rarely occurs in a normal healthy eye, due to the human cornea’s natural resistance to infection. However, contact lens wear, corneal surgery, trauma, ocular surface disease, and systemic disease, such as diabetes mellitus or immunosuppression, are predisposing factors associated with increased risk of infection [7]. Identification of virulence factors and the host response to the invading bacterium are critical to understanding the disease process in order to develop effective treatment modalities.

Virulence of the invading organism depends on its ability to penetrate and colonize the cornea, resist host defense mechanisms, and produce corneal damage [8]. Colonization of the host cells is mediated by adhesins expressed on the bacterial surface that bind to receptors on the host cell surface. Adhesins may also act as toxins [9]. Many bacteria display several adhesins on fimbriae and non-fimbriae structures. These adhesive proteins recognize carbohydrates on host cells and bind to these cells via protein-protein interactions. Tissue damage is usually mediated by exogenous proteins secreted by the bacterium or secondary effector molecules that assist in the infective process. Upregulation or downregulation of host defense mechanisms may also be involved. Some bacteria such as *S. aureus*, *S. pneumoniae*, and *P. aeruginosa* adhere to ulcerated corneal epithelium at relatively higher rates than other bacteria, making them the most commonly isolated organisms [10].

Although many virulence factors have been identified thus far using traditional approaches such as cloning, polymerase chain reaction (PCR), gene knockout, and antisense technology, proteomic methods such as enzyme-linked immunosorbent assay (ELISA), antibody arrays and Western blotting used in combination with these approaches have contributed enormously to our current knowledge of the pathogenesis of keratitis. This short review paper will discuss the use of the few proteomic approaches used to date for the study of bacterial keratitis, including identification of virulence factors and bacteria-host interactions for the most frequently isolated organisms. Furthermore, we will address the value of expanding these studies and the need for more global proteomic approaches to the study of bacterial keratitis.

## 2. Staphylococcus Aureus

*S. aureus* is one of the most significant pathogens in bacterial keratitis [11,12]. Its incidence varies worldwide, but its increased resistance to certain antibiotics makes it an important global healthcare issue [13,14]. *Staphylococcal* keratitis is characterized by destruction of the cornea via bacterial exoprotein deposition and the host inflammatory response to infection [15]. Although antibiotic therapies may succeed in reducing or eliminating the bacterial load, damage from scarring, loss of visual acuity and blindness may still result. Additionally, the emergence of multidrug-resistant *S. aureus* strains further complicates therapeutic strategies [16].

The mechanisms involved in the initiation of *S. aureus* keratitis are not yet understood. *S. aureus* has been shown to adhere to corneal epithelial cells via fibronectin and collagen [17,18,19]. Virulence factors produced by *S. aureus* in keratitis include α-toxin as the major factor, with β and γ-toxins to a lesser extent [15,20,21]. O’Callagan *et al.*, used proteomic approaches including polyacrylamide gel electrophoresis (PAGE), Western blotting, silver staining and enzyme assays to purify the α and β-toxins and assess their ocular toxicity in New Zealand white rabbits [15]. These studies confirmed the contribution of α-toxin to ocular damage and identified the role of β-toxin in keratitis. The administration of purified α-toxin was found to directly destroy the epithelium, and mutants deficient in α-toxin caused less corneal edema than their isogenic parent strains. For the β-toxin, its administration to the eye demonstrated that it can mediate edema in the sclera and conjunctiva. This suggested that the two bacterial proteins identified, α-toxin and β-toxin, could be targeted for a new type of chemotherapy designed to limit the ocular damage caused by these toxins and reduce the major tissue damage and scarring reactions associated with *Staphylococcus* keratitis.

In order to understand the contribution of the host response to *S. aureus* infection, a keratitis mouse model was developed in both C57BL/6 and BALB/c mice using both virulent and non-virulent strains of *S. aureus*. Using ELISA, the authors detected significant upregulation of IL-4, IL-10, IL-6, and macrophage inflammatory peptide (MIP)-2 in the mice infected with the virulent strain [22]. The author suggested that IL-4, IL-10 and IL-6 cytokines may be protective during Gram-positive corneal infection and therefore, may be useful as adjunct therapies during treatment.

In an effort to test the efficacy of antimicrobials on a co-culture of the bacterium *S. aureus* and the fungus *Fusarium solani*, a recent study used 2D gel electrophoresis and Matrix-Assisted Laser Desorption/Ionization Time-of-Flight (MALDI-TOF) to study the proteomic profile of the co-culture with and without antimicrobials*.* In the presence of antimicrobials, *S. aureus* and *F. solani* were found to interact and the co-culture showed differential protein expression when grown without antimicrobial agents [23]. This may suggest that the bacterial-fungal interaction affects protein expression and pathogenesis.

## 3. *Pseudomonas Aeruginosa*

**Figure 1:**
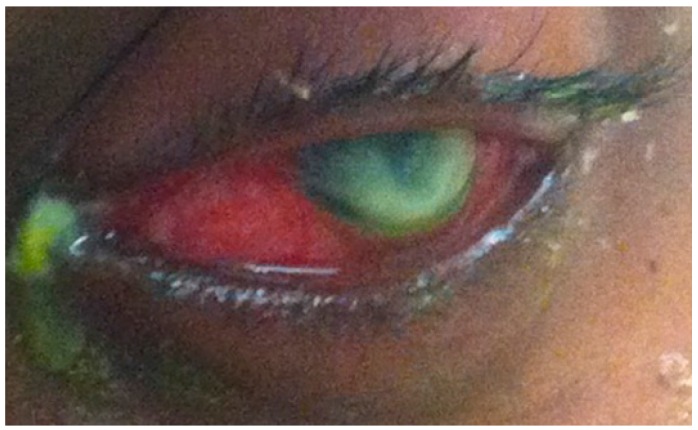
A photograph showing a bacterial keratitis infection caused by *P. aeruginosa.*

*Pseudomonas aeruginosa* (*P. aeruginosa*) is a ubiquitous Gram-negative bacterium associated with bacterial keratitis and one of the most destructive among opportunistic pathogens. *P. aeruginosa* keratitis progresses rapidly and is characterized by infiltration of inflammatory cells (Figure 1) and tissue destruction leading to corneal perforation [24]. Recent reports confirm that *P. aeruginosa* is the most commonly isolated organism from contact lens wearers, the group with highest risk for keratitis infection [4,25,26]. In 2002, it was reported that 25,000–30,000 contact lens wearers developed microbial keratitis annually in the United States [27], and that 6%–39% of the cases are caused by *P. aeruginosa* [28,29]. Currently, there are at least 34 million contact lens users in the United States and 140 million worldwide [30].

The pathogenesis of *P. aeruginosa* is mediated through a plethora of virulence factors. These include cell-associated structures, such as pili [31] and flagella [32], and extracellular products, such as alkaline protease [33], elastase B (LasB) [34], exoenzyme S [9], exotoxin A [35], endotoxin [36], slime polysaccharide, phospholipase C, leukocidin, protease IV [37], and *P. aeruginosa* small protease (PASP) [38]. Whereas Gram-positive bacteria, including *S. aureus*, adhere to host tissues via fibronectin and collagen [17], *P. aeruginosa* attach to cell surfaces that lack fibronectin [39]. *P. aeruginosa* adhere to injured cornea [40], exposed corneal stroma [41], or immature non-wounded cornea [42]. The corneal epithelial receptors for *Pseudomonas* spp. have been identified as glycoproteins [43]. SDS-PAGE and Western blot analysis were performed on purified PASP and LasB to determine their role in keratitis [38].

An important factor that contributes to the destruction of the cornea during bacterial keratitis is excessive activation of the host defense system. *P. aeruginosa* can activate several pathways of the immune system during infection, and activation often involves toll-like receptors (TLRs) on the corneal epithelium. These TLRs recognize lipopolysaccharides or flagella from *P. aeruginosa* and activate the epithelial cells to produce inflammatory mediators such as cytokines and chemokines. These cytokines or chemokines recruit white blood cells, predominantly polymorphonuclear leukocytes, to the site of infection in order to eliminate the *P. aeruginosa*. Karthikeyan *et al.* examined corneal ulcers from patients with P. aeruginosa and found elevated expression of the pathogen recognition receptors TLR2, TLR4 and TLR9, pro-inflammatory cytokines IL-1α, IL-1β, and IFN-γ, and the inflammasome components NLRP3, NLRC4 and ASC compared with donor corneas [44]. Because neutrophils were the predominant cell types in these corneal ulcers, the author suggested that they may be the source of the majority of these factors. The authors used Western blotting to characterize the exotoxins (ExoS, ExoT and ExoU) secreted by *P. aeruginosa* clinical isolates [44]*.* Using a *P. aeruginosa* keratitis mouse model, several ELISA studies have shown increased IL-1β, IL-6, IFN-γ, TNF-α, and IL-12 p40 compared to uninfected control eyes [45,46]. The kinetics and identities of inflammatory cytokines produced were found to be bacterial strain- and time-dependent [47,48,49]. The role of IL-12 in ocular *P. aeruginosa* infection has also been explored using ELISA and immunocytochemistry in combination with standard molecular techniques [50].

Protein arrays have also been used to study *P. aeruginosa* keratitis. Sack *et al.* used this approach to delineate the spectrum of angiogenic bioactive protein modulators that might be secreted and upregulated by the corneal epithelium in response to killed *P. aeruginosa* products and revealed that the immortalized cell lines constitutively secrete several proteins and upregulate secretion of IL-6, IL-8, and GRO in response to killed bacteria [51]. These studies revealed the role of innate and adaptive immune defense system in keratitis.

Recently, Sewell *et al.* [52] performed a global proteomic approach, using liquid chromatography followed by tandem mass spectrometry, to compare a clinical isolate of *P. aeruginosa* from an active corneal ulcer with a non-pathogenic laboratory strain of *P. aeruginosa* (ATCC10145) and found a total of 133 proteins that were significantly different between the two strains. The upregulated proteins were associated with virulence and pathogenicity [52] and included flagellin B, lipotoxin F, organic solvent tolerant protein, polyhydroxyalkanoate synthesis protein and dehydrocarnitine CoA transferase subunit B. In addition, two putative nonribosomal peptide synthetases (NRPS) were detected in the corneal strain but not ATCC10145. The NRPSs are responsible for the production of the secondary metabolite l-2-amino-4-methoxy-*trans*-3-butenoic acid (AMB), a potent toxin produced by *P. aeruginosa* [52]. This suggests that *P. aeruginosa* might be using AMB as a virulent factor in keratitis. Further studies are warranted to confirm this hypothesis.

## 4. *Streptococcus Pneumoniae*

*S. pneumoniae* (pneumococcus) is also a common cause of infectious keratitis after *P. aeruginosa* and/or *S. aureus* [11,29,53,54,55,56,57,58]; however, some epidemiologic studies identify it as the top cause of bacterial keratitis [59,60,61,62,63,64,65]. Unlike *P. aeruginosa*, pneumococcal keratitis is not typically associated with contact lens use but rather with predisposing conditions such as ocular trauma or surgery [53,58,59,60,63,65,66,67,68,69].

The outer capsule of *S. pneumoniae*, composed of polysaccharide necessary to establish virulence and survive the host immune response, is the most studied virulence factor for this bacterium [70,71]. With infections, such as pneumoniae, otitis media, meningitis and septicemia, the noncapsular forms of bacteria are avirulent. Whereas with keratitis, noncapsular strains have been shown to cause as severe keratitis as their capsular counterparts in intrastromal infection models [72,73].

Another virulence factor, pneumolysin (PLY), is a pore-forming toxin that was first identified by Johnson and Allen [74] as being responsible for ocular tissue damage during pneumococcal keratitis [74,75,76]. This toxin causes both direct cellular damage by forming pores in host cell membranes and immune-derived damage by activating the complement system and inducing inflammation. It has also been found that PLY reduces the opsonic activity of S. pneumoniae, which could allow for more bacterial replication and more toxin release [77,78]. Proteomic approaches have been used to determine the structure and function of PLY [79,80,81], as well as to investigate whether passive immunization with pneumolysin antiserum could reduce corneal damage associated with pneumococcal keratitis [82]. These studies used ELISA, Western blotting and purification of recombinant PLY and found that passive immunization with antiserum to PLY can significantly minimize the initial corneal damage commonly observed with pneumococcal keratitis and promote full recovery from keratitis. The finding suggests a novel treatment for pneumococcal keratitis by using antibodies to PLY, or peptides constructed of antibody epitopes, in addition to the customary antibiotic therapy [82]. No other protein contributing to virulence in pneumococcal keratitis has been identified.

As for the host response in corneal ulcers from patients with culture positive S. pneumoniae, similar to *P. aeruginosa*, Karthikeyan *et al.* also found elevated expression of TLR2, TLR4, TLR9, IL-1α, IL-1β, IFN-γ, NLRP3, NLRC4 and ASC compared with control non-infected corneas. The authors used Western blotting to confirm the expression of PLY in *S. pneumoniae* clinical isolates [44]*.*

## 5. *Serratia* Species

*Serratia* species are opportunistic Gram-negative bacteria that belong to the large family of Enterobacteriaceae, with *Serratia marcescens* being the primary pathogenic species [83]. Risk for *Serratia* keratitis is associated with abnormal corneal surface, topical medication use, and contact lens wear [5,84]. It can also cause refractory keratitis resulting in corneal perforation and blindness.

*S. marcescens* produces four different proteases, as well as two nucleases [85,86], all of which were isolated and characterized using proteomic approaches such as protein precipitation, isoelectric focusing and gel electrophoresis [85]. One protease of 56 kilodaltons (56K protease), which is the major pathogenic factor in Serratial keratitis [87], was purified from the culture supernatant of a strain of *S. marcescens* isolated from a severe corneal ulcer of a human eye. Purification of this protease was achieved using different proteomic methods that included ammonium sulfate precipitation, DEAE-cellulose ion-exchange chromatography, Sephadex gel filtration chromatography, polyacrylamide gel electrophoresis and immunodiffusion [87]. The 56 kDa protease activates Hageman factor, initiating the Hageman factor-kallikrein-kinin cascade, which leads to enhanced vascular permeability [88,89]. This study also used chromatography techniques for purification of 56K protease and Hageman factor. Subsequently, the production and purification of an anti-56K protease antibody that was used for immunization purposes [89]. The protein structure of the 56 kDa protein was recently determined by Bhaskar *et al.* [90]. These studies were performed to determine the role of the 56 kDa protein in the pathogenesis of serratial infections in the eye as well as confirming the inflammatory process in the infection. To study the host response to serratial infections of the cornea, Zhou *et al.* also used ELISA to measure cytokine production in infected corneas as well as in the supernatants of stimulated bone marrow-derived macrophages confirming an inflammatory process in serratial keratitis [91].

## 6. Other Studies

### 6.1. Host Response Studies

Multiple laboratories have studied the host response mechanisms to corneal infection by different bacteria, some of which are mentioned in the above sections. Using keratitis animal models and ELISA, these studies investigated the inflammatory processes and the immune response of the host to the infection [92,93,94,95,96,97,98,99,100,101,102]. Although, the majority of these studies were focused on pseudomonas keratitis, findings from other studies of different bacteria were similar. All studies confirmed the role of toll like receptors (TLR 2, 6, 4 and 9), interleukins (IL-8, IL-18, IL-6, and IL-1β, IL-10, IL-17) and metalloproteinases (MMP-9) in addition to NFκB, TNF-α*,* JNK, and p38 among other proteins in bacterial keratitis. In addition, because of their role in clearance of debris and pathogens from the surface of the eye and protection against infections, secreted mucins and their O-glycans in the tear film have been studied, and their protein structure has been elucidated [103,104].

### 6.2. Contact Lens Studies

Because contact lens wear is a major risk factor for bacterial keratitis, studies to investigate the mechanisms of host responses to infections resulting from contact lens wear [105,106] were conducted. These studies investigated contact lens protein deposits. Zhao *et al.* used liquid chromatography combined with tandem mass spectrometry (LC-MS-MS) and found that the worn contact lenses contained a wide array of proteins deposited from tear film and other sources and that these protein deposit profiles were varied and specific for each lens material tested [106]. Green-Church *et al.* used a similar approach, nano-liquid chromatography tandem mass spectrometry (nano-LC-MS/MS), to investigate the proteomes of two daily wear silicone hydrogel contact lenses when used with two multipurpose care solutions [105]. The authors reported that the contact lens protein deposition profiles showed a high degree of similarity between the two silicone hydrogels, consisting mainly of six proteins including lipocalin, lysozyme, lacritin, lactoferrin, proline rich 4, and Ig-Alpha. However, some unique proteins were also detected for each polymer type, not only providing valuable information about the tear film proteome itself but also yielding insight about the interaction between these polymers and tear film proteins [105]. These studies were conducted in an effort to determine the optimal contact lens material because it has been estimated that 80% of clinical issues and 30% of aftercare visits relating to extended wear of conventional contact lenses were due to deposition of tear-derived substances on the lens surface [107]. However, a recent comprehensive review suggests that deposition of proteins, such as lysozyme and lactoferrin, on contact lens materials may actually be beneficial during contact lens wear, as these proteins can reduce the viability of bacteria on the contact lens, thus slowing or preventing the pathogenesis of contact lens-related microbial keratitis and inflammation [108].

## 7. Concluding Remarks and Future Perspectives

In addition to the identification of virulence factors, the studies described above have helped to increase our understanding of the innate immune system’s ability to recognize invading bacteria at the corneal surface through Toll-like receptors (TLRs) expressed on the surface of epithelial cells, macrophages, and dendritic cells in the stroma. These TLRs recognize conserved bacterial surface proteins, such as lipopolysaccharide (LPS), leading to rapid production of proinflammatory and chemotactic cytokines and recruitment of neutrophils to the corneal stroma. This process seems to be common to all bacteria described. These studies led to the development of current therapies and the design of better contact lens materials. However, more studies are needed to further characterize the mediators of innate and adaptive immunity, as well as to identify other virulence factors that could potentially be used as targets for novel therapies. In addition, studies to understand mechanisms of bacterial resistance are also needed. All these can be studied using proteomic approaches. Proteomic analysis has been used in medical microbiology for the identification of novel pathogenic mechanisms, investigation of the epidemiology and taxonomy of microbial pathogens, the analysis of drug resistance [109], and in the design and development of antimicrobial vaccines [110]. In each of these areas, proteomics has provided new insights that complement genomic-based investigations, as a genomics approach alone is typically insufficient. As mentioned in this review, most studies of bacterial keratitis to date have used standard molecular techniques, such as cloning, PCR, and gene knockout while very few have incorporated a proteomic approach. Although limited, the few proteomic studies described in this short review have helped tremendously to gain insight into the pathophysiology of keratitis; however, compared to studies performed on fungal or viral keratitis, these studies were limited. There is an urgent need for more proteomic studies such as those performed by Sewell *et al.*, comparing a corneal strain of *P. aeruginosa* to a non-corneal/non-pathogenic laboratory strain [52] by Hare *et al.* in the study of cystic fibrosis [111], and those performed to study the tear proteome in fungal keratitis [112,113]. Global proteomic methods such as Liquid Chromatography/Tandem Mass Spectrometry (LC-MS/MS) have been widely used for molecular characterization of diseases [114,115,116,117]. Global comparisons of clinical and non-clinical strains would undoubtedly reveal novel virulence factors and aid in the diagnosis, prognosis and treatment of bacterial keratitis. Finally, studies focusing on the functional properties of proteins implicated in the pathogenesis of bacterial keratitis may also contribute to a better understanding of the disease and development of new drugs for treatment.
